# Three-color single-molecule localization microscopy in chromatin

**DOI:** 10.1038/s41377-025-01786-1

**Published:** 2025-03-17

**Authors:** Nicolas Acosta, Ruyi Gong, Yuanzhe Su, Jane Frederick, Karla I. Medina, Wing Shun Li, Kiana Mohammadian, Luay Almassalha, Geng Wang, Vadim Backman

**Affiliations:** 1 Department of Biomedical Engineering, Northwestern University, Evanston, IL 60208 USA; 2 Center for Physical Genomics and Engineering, Northwestern University, Evanston, IL 60208 USA; 3 IBIS Interdisciplinary Biological Sciences Graduate Program, Northwestern University, Evanston, IL 60208 USA; 4 Applied Physics Program, Northwestern University, Evanston, IL 60208 USA; 5 Department of Gastroenterology and Hepatology, Northwestern Memorial Hospital, Chicago, IL 60611 USA

**Keywords:** Applied optics, Imaging and sensing, Super-resolution microscopy

## Abstract

Super-resolution microscopy has revolutionized our ability to visualize structures below the diffraction limit of conventional optical microscopy and is particularly useful for investigating complex biological targets like chromatin. Chromatin exhibits a hierarchical organization with structural compartments and domains at different length scales, from nanometers to micrometers. Single molecule localization microscopy (SMLM) methods, such as STORM, are essential for studying chromatin at the supra-nucleosome level due to their ability to target epigenetic marks that determine chromatin organization. Multi-label imaging of chromatin is necessary to unpack its structural complexity. However, these efforts are challenged by the high-density nuclear environment, which can affect antibody binding affinities, diffusivity and non-specific interactions. Optimizing buffer conditions, fluorophore stability, and antibody specificity is crucial for achieving effective antibody conjugates. Here, we demonstrate a sequential immunolabeling protocol that reliably enables three-color studies within the dense nuclear environment. This protocol couples multiplexed localization datasets with a robust analysis algorithm, which utilizes localizations from one target as seed points for distance, density and multi-label joint affinity measurements to explore complex organization of all three targets. Applying this multiplexed algorithm to analyze distance and joint density reveals that heterochromatin and euchromatin are not-distinct territories, but that localization of transcription and euchromatin couple with the periphery of heterochromatic clusters. This work is a crucial step in molecular imaging of the dense nuclear environment as multi-label capacity enables for investigation of complex multi-component systems like chromatin with enhanced accuracy.

## Abstract (web summary)

We present a protocol enabling multi-color chromatin SMLM, revealing transcription and enhancer-associated euchromatin organizes around heterochromatic cores into functional volumes. This significantly advances nanoscopic molecular imaging within the nucleus.

## Graphical abstract

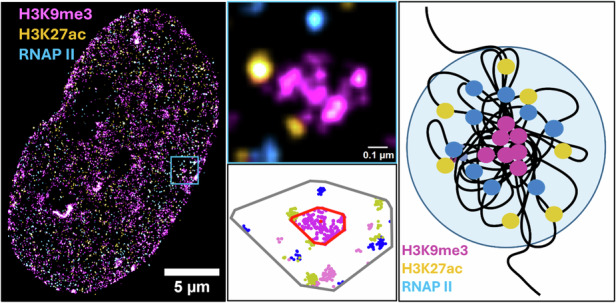

## Introduction

The development of single-molecule localization microscopy (SMLM) has allowed unprecedented investigation of the nanoscopic structure of biological tissues^1–5^. These capabilities have been greatly enhanced by the capacity to image multiple molecular species, allowing the investigation of the relationship between sub-diffraction structures in space and time^6–11^. Among the most complex structures within cells is chromatin, the assembly of the genome folded into three-dimensional space^12–14^. With the rapid advancement of sequencing-based methods (chromatin immunoprecipitation sequencing, high-throughput chromatin conformation capture, etc.)^15–19^, several models describing chromatin folding such as the loop extrusion model^20–22^ have been proposed to recapitulate the findings from inferred models through studies on topologically associated domains (TADs) and genome compartments^15,21–23^. SMLM studies, based on DNA-Point accumulation for imaging in nanoscale topography (DNA-PAINT) and multiplexed-fluorescence in situ hybridization^15,16^, utilized the inferred localization of molecular labels obtained by these methods to provide information about the underlying structure of the genome upon imaging^23,24^. However, these methods rely on formamide denaturation which can result in disruption in the underlying space-filling conformations of the genome in space^25^. Therefore, it was not until the advent of chromatin electron microscopy (ChromEM)^23^ that the ground truth for the in vitro sub-diffraction supra-nucleosome genome structure was observed, remained elusive at the regulatory length-scales that were beyond conventional imaging modalities (<200 nm)^26,27^.

As described by Ou et al.^28^, chromatin is organized as a disordered polymer at the smallest length scales, ranging from 5 to 20 nm. Utilizing the ChromEM framework and high-angle annular dark-field imaging technique, chromatin scanning transmission electron microscopy (ChromSTEM)^29^ allows for the identification of higher-order chromatin structures (packing-domains) at supra-nucleosome length-scales with a distribution of sizes and length-scales present without a characteristic partitioning into discrete heterochromatin (compacted) and euchromatin (accessible) states^30,31^. Given this surprising finding that interphase chromatin is not assembled into discrete functional territories, it is imperative to develop new methods of analysis and imaging of chromatin conformation to understand the molecular structure of supra-nucleosomal folding. At length-scales between 100 and 200 nm, Miron et al.^27^ and Li et al.^32^ described how heterochromatin and euchromatin coupled to produce the complex formation of packing-domain (PD) structures. Using structured illumination microscopy (SIM), Miron et al. found that heterochromatin is organized within PD centers, with euchromatin and RNA-polymerase extending outward to the periphery at a label localization precision of 80–100 nm. Li et al. using a combination of single color SMLM of active RNA polymerase and partial-wave spectroscopic (PWS) microscopy^33,34^, which, although diffraction limited, is sensitive to folding of structures between 20 and 200 nm, found that active RNA polymerase localizes to the boundary of PDs^32^. Despite this finding, there remains a crucial gap in our understanding of the molecular assembly of the genome at length-scales between 20 and 100 nm. Indeed, recent work utilizing ChromSTEM tomography has shown that packing domains are heterogeneous structures with variations in assembly and density at length-scales ranging from 50 to 200 nm^35^.

Consequently, the development of multiplexed SMLM to study the spatial relationship between heterochromatin, euchromatin, and RNA polymerase at higher resolution, multi-color imaging could uncover new molecular processes in the regulation of genome structure. Multiplexed SMLM, including multi-color direct STORM (dSTORM)^9^ and (DNA-PAINT)^36^, has elucidated multiple targets and their spatial relationships in single cells with resolution up to 10 nm^36^. dSTORM utilizes immunostaining, while DNA-PAINT employs fluorophore-bound imager DNA strands that bind to its complementary docking strands attached to specific targets. Recently reported DNA-PAINT achieved labeling of up to 30 targets within cells^23^, but the denaturation-based probe binding disrupts chromatin organization by breaking down the double-strand structure of DNA with formamide^25^. To elucidate the chromatin domain structure while preserving its integrity, immunostaining-based super-resolution, such as dSTORM, imaging remains the only viable solution so far. However, due to the dense packing environment in chromatin, three-color dSTORM for nuclear targets has never been reported although two-color chromatin dSTORM and three-color dSTORM in cytoplasm have been documented.

Adding to the complexity of genome structure is the fact that it is inherently a disordered polymer structure, therefore its reconstruction from sparse emissions contrasts with the capacity to resolve simpler structures such as microtubules as the distribution of emission events requires more complex spatial analysis. Likewise, probe targeting of highly dense chromatin polymer with volume concentrations between 0.2 and 0.8 is challenging, which requires a high label density, but the accessibility of chromatin is poor, especially to the large labels required for molecular recognition. This is compounded by difficulties in delivering high label concentrations and ensuring their diffusion through the dense nuclear environment^37–42^. Moreover, label density, together with photon-budgeted localization defines spatial resolution, which is limited to twice the spatial distance between labels. Currently, the reported maximum number of colors used in chromatin super-resolution imaging remains limited to two^43–46^. Besides the prior issue, more challenges exist to imaging and analysis of chromatin at length-scales below 200 nm utilizing SMLM methods: (1) Staining protocol, imaging buffer, fluorophore stability, and antibody specificity must be optimized to achieve proper antibody conjugates. (2) Complex analysis of multiple labels is required as the relationship between euchromatin and heterochromatin with enzymes such as RNA-polymerase may be more complex than binary exclusions.

In this work, we developed a 3-color chromatin SMLM in vitro overcoming the challenges of multi-labeling in a dense structure by optimizing antibody conjugation timing and utilizing newly described imaging buffers^44^. To facilitate the complex analysis of 3-color SMLM, we develop a clustering-based 2-color distance analysis and 3-color joint density analysis methods to compare multiple coupled molecules within these confined spaces. We demonstrate that in contrast to prior work with 2-color chromatin SMLM showing the separation of heterochromatin and euchromatin into two partitions, three-color chromatin imaging demonstrates that the genome organizes into packing domains with euchromatin and active transcription occurring around the periphery of constitutive heterochromatin cores. This work presented a method to reliably achieve multi-color SMLM in chromatin to probe additional complex interactions with a data analysis methodology to probe the complex interactions of the genome.

## Results

### Sequential labeling and optimized imaging buffer facilitate three-color single-molecule localization microscopy of heterochromatin, euchromatin, and active RNA polymerases

**Fig. 1:**
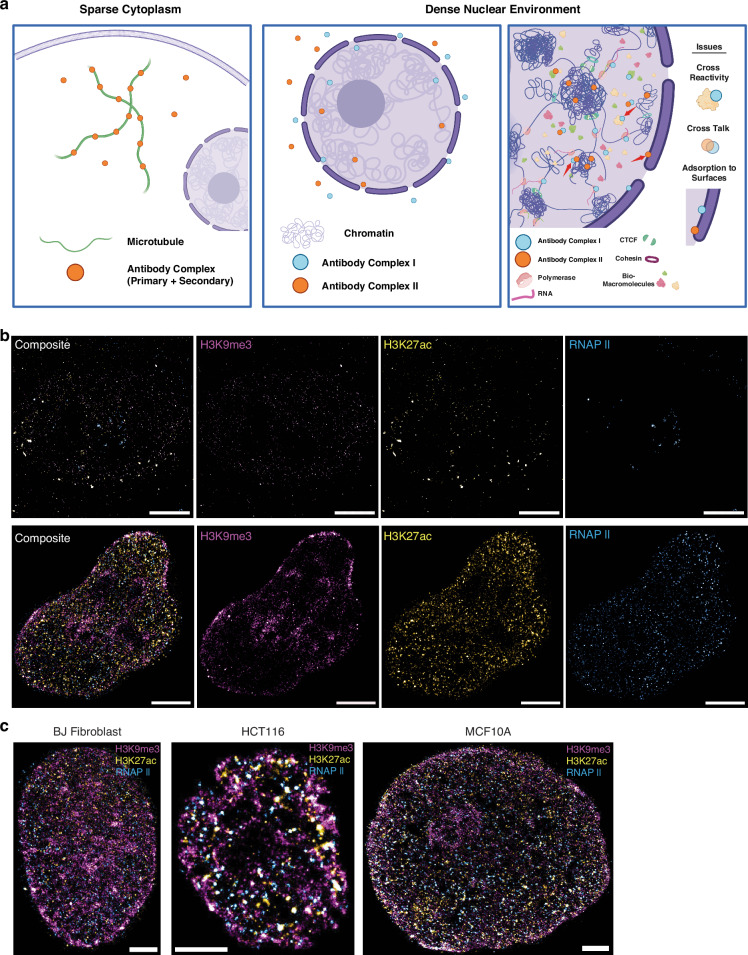
Unsuccessful and successful staining for 3-color SMLM in multiple cell lines. **a** Schematic showing the difficulties of multi-labeling in a dense nuclear environment relative to sparse cytoplasm. The first panel showcases microtubules being labeled by antibody complex (primary and secondary are shown as one circle for clarity in the diagram). In a sparse environment, the antibodies are not hindered by neighboring structures and can reach their target. In the middle panel, we showcase the difficulty of getting antibodies in the nucleus, with some reaching the interior and labeling chromatin, while the majority lies outside the nuclear envelope. The third panel illustrates the issues of labeling a target within the dense nucleus: cross-reactivity shown by Antibody Complex I (which in this example aims to label RNAPII) reacting with other nuclear macromolecules, cross talk due to proximity, and adsorption to non-target surfaces due to high crowding. We have added red arrows to showcase these examples within the diagram. **b** Top row shows the failing staining of H3K9me3, H3K27ac, RNA Polymerase II in OVCAR5 cells, based on simultaneous staining protocol, and their merge image respectfully, while **b** bottom row displays the successful staining by sequential staining protocol in HeLa cells. All images scale bar is 5 µm. **c** Exemplary three-label SMLM images targeting H3K9me3, H3K27ac, and RNAPII for BJ Fibroblast, HCT116, and MCF10A cell lines. All scale bars are 3 µm

There are multiple different approaches for targeted binding of immunofluorescent markers in super-resolution microscopy. Among the most widely used is the concurrent incubation of primary antibodies against multiple targets with the subsequent addition of concurrent secondary antibodies. As each antibody is derived from a different host organism, it is generally assumed that their binding efficiencies will result in the binding of the distinct targets concurrently. Therefore, we first applied the published staining protocol for 3-color SMLM labeling which was originally for Tom20, ATPB, and tubulin^9^, to staining of chromatin markers associated with the differential states of genome function: H3K9me3 (constitutive, high-density heterochromatin), H3K27ac (enhancer associated euchromatin) and transcriptionally active RNA polymerase II (RNAP II)^9^. In this protocol, all three primary antibodies are incubated with the cells simultaneously during the primary antibody incubation, followed by simultaneous incubation of all three secondary antibodies during the secondary antibody incubation. Images show that all three channels are sparsely labeled with extremely low localization density (Fig. 1b Top, Fig. S3c). In the loosely organized cytoplasmic environment, the accessibility of antibodies to their targets is relatively unimpeded. In contrast, within the densely packed nuclear environment, the presence of diverse macromolecules, such as polymerases and RNA, occupies the limited space, thereby hindering the ability of antibodies to effectively bind to their corresponding targets (Fig. 1a). Although simultaneous incubation can successfully stain structures in the lower density cytoplasm^9^, the highly dense nuclear region can non-monotonically influence molecular interactions and lead to non-specific binding, such as histone markers and active RNA polymerases. Therefore, we developed a sequential labeling method (Fig. S1b), in which the cells are only incubated with one type of antibody at a time to allow sufficient space for antibody binding before moving to the next antibody incubation. Between each labeling sequence (the washing step is not noted in Fig. S1b), samples underwent repeat blocking to minimize the frequency of non-specific binding and adsorption^47^. The choice of blocking with goat serum was derived from the secondary antibody source, which blocks the attachment of Fc receptors in the sample to both the primary and secondary antibodies used in experiments^48^.

The fluorophores we used for the 3-color SMLM were AF647, AF568 and AF488. During the imaging process, we excited the fluorophores, respectively, by 637, 532, 488 nm laser lines in sequence to achieve minimum inter-channel photobleaching caused by blue light. The sequential imaging process usually took 8–10 min to obtain 10,000 frames for each channel. In total, one 3-color SMLM image took around 25 min. As a result, one limitation of multiplexed SMLM imaging is the collection of multiple cells in different conditions might take up to 4 h or even longer. Achieving sustained emission efficiency across such a timeframe and multiple fluorophores requires careful selection of SMLM buffers. To achieve this end, we utilized a recently described optimized imaging buffer that does not rely on the catalase reduction system^47^. Despite the extended sample preparation time of 3 days for staining, this sequential imaging protocol successfully achieved 3-color SMLM for targets in the nuclear region of multiple distinct functions and molecular states. We have demonstrated its applicability in multiple cell lines, including HeLa, BJ fibroblast, HCT116, and MCF10A (Fig. 1b bottom, 1c). Localization estimates were generated using the Thunder-STORM Image J plugin^49^ with the maximum likelihood estimation algorithm and peak intensity threshold weighting coefficient of 2–3. The average uncertainty of localizations varies from 10 to 20 nm (Fig. S2). We also calculated the multi-blink property of these three fluorophores in the optimized imaging buffer using a spectral regression method adapted from what was described in Dong et al.^50^ (Fig. S3a).

### Simulation of different statistical distributions validate the point cloud-based and image-based methods for analysis multi-color SMLM data

**Fig. 2:**
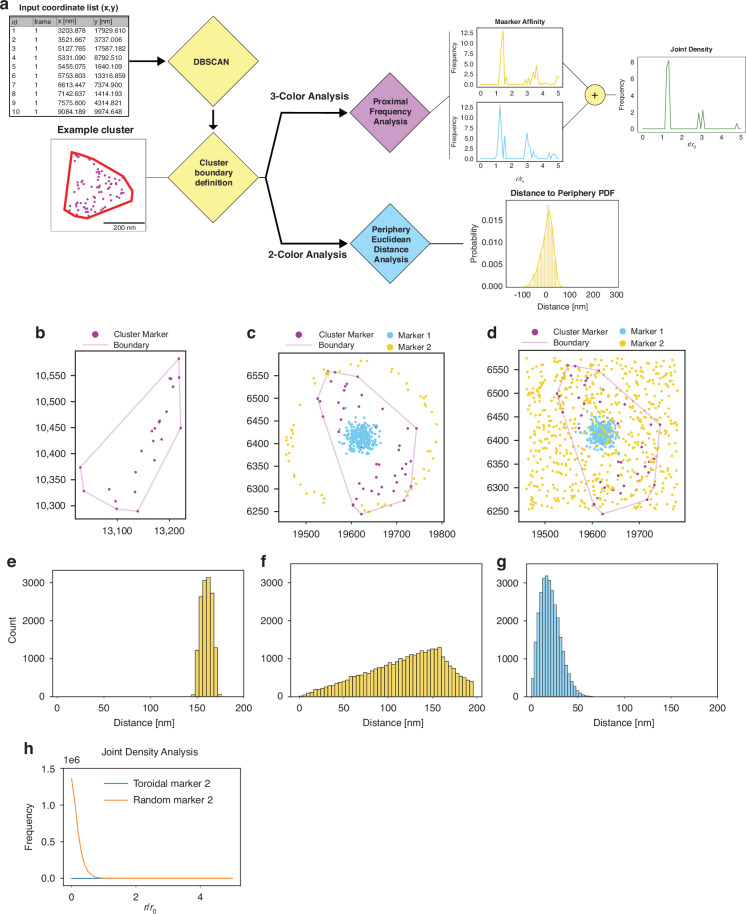
Simulated 2-color and 3-color results for point-cloud based algorithms demonstrate algorithm robustness. **a** Shows our proposed pipeline for point-cloud-based analysis of 2 and 3 color SMLM datasets. **b** Showcases an example heterochromatic cluster with fitted cluster periphery. **c** Shows example one of two simulated spatial distribution cases (Gaussian for Marker 1 in blue and Toroidal for Marker 2 in yellow) overlayed an example heterochromatin cluster with periphery fitted**. d** Demonstrates the second simulation case similar to (**c**) but with a random spatial distribution for simulated Marker 2 in yellow. **e–g** The 2-label spatial distance count histogram results for the point-cloud-based algorithm with the same distributions demonstrated in (**c**) and (**d**). **h** Shows the joint density curves for the two test cases shown in (**c**) and (**d**), respectively, with simulation case (**c**) corresponding to the blue curve and simulation case (**d**) corresponding to the orange curve. Joint Density curve shown is relative to the heterochromatin centroid

Following the successful implementation of the 3-label immunofluorescence labeling protocol, we developed analysis pipelines to investigate the molecular and functional composition of chromatin in multiplexed samples (Figs. 2a and S6). SMLM datasets can be analyzed using either point-cloud-based methods that directly utilize localizations or image-based approaches that work with reconstructed images^26,51^. While both approaches were developed (Figs. 2a, S6 and detailed Supplementary Information), we focus on point-cloud methods as they provide direct analysis of spatial relationships without potential artifacts from image reconstruction and binning. The point-cloud approach allows for more granular spatial analysis that avoids binning when working with biological datasets, enabling precise measurements of distances between different molecular and functional components with a myriad of established clustering analysis methods^48,49^. This strategy was pursued due to the limited knowledge of the ground-truth structure of chromatin at these length scales, where analyzing the spatial relationships between different molecular and functional components provides insights into nuclear organization.

To elucidate the spatial relationships between heterochromatin and other nuclear components, we employed density-based spatial clustering of applications (DBSCAN)^26,51^ for its demonstrated ability to handle varying density distributions and non-spherical clusters, making it particularly suitable for analyzing the complex arrangements of nuclear targets in dense environments^52^. This clustering approach enables the identification of domains without imposing predetermined size or shape constraints. We selected H3K9me3 as our seed point based on prior super-resolution studies showing the concentric distribution of heterochromatin to euchromatin within the nucleus at the 200 nm length scale^27,29^. Clustering parameters epsilon and minimum points were determined via a customized grid search algorithm which used a domain shape score to evaluate clusters and monitor clustering efficacy (Fig. S4f and Supplementary Information). Following cluster identification, we implemented Convex Hull estimation to define domain boundaries based on observed localizations rather than imposing predetermined geometries. This approach enables analysis of spatial relationships between different chromatin states and functional elements while avoiding assumptions about domain structure where no localizations are present (Fig. 2b). For a more detailed description of all steps of our analysis method please refer to the Supplementary Information.

With established cluster identification, we probed the performance of our analytical methods with different spatial distributions before applying it to biological datasets, we tested it using simulated localization datasets that follow distinct statistical patterns relevant to nuclear organization. We employed these distributions (Gaussian, Random, Uniform, Toroidal) as they are expected to be easily distinguishable from each other when measuring the spatial distance to our relative seed points (Fig. S7a). The seed points were determined from actual heterochromatic datasets to mimic observed distributions and not assume spacing between clusters a priori (Fig. 2b). Our analysis produced visually distinct distance histograms for each tested distribution (Figs. 2e–g and S7c), demonstrating its capability to differentiate various spatial relationships between labeled components. This validation was crucial as it confirmed our ability to detect different organizational patterns of chromatin states and functional elements, even without knowledge of the underlying structure. In addition to our Euclidean distance metric, we employed our developed joint density analysis which aims to uncover areas of enrichment for both other labels relative to the landmark seed points, providing insights into spatial relationships that cannot be captured through pairwise analysis alone. We validated this approach by examining cases where one marker maintained a constant Gaussian distribution while the second marker followed either toroidal or random distributions (Fig. 2c, d). In the Gaussian-toroidal case where distributions did not overlap, we observed the expected flat distribution within the analysis region (Fig. 2h). Conversely, the Gaussian-random case with direct marker overlap demonstrated a decaying distribution with joint density decreasing with distance from cluster centroids. These simulation results validate our analysis framework’s ability to distinguish different spatial relationships between labeled targets, providing confidence in its application to biological datasets where we probe the molecular composition and functional organization of chromatin domains. This analysis framework enables systematic investigation of spatial relationships between heterochromatin domains and other chromatin states or functional elements at length scales previously inaccessible to diffraction-limited techniques.

### Enhancer-associated euchromatin and active RNA Polymerase II localize at the periphery of heterochromatin clusters

**Fig. 3:**
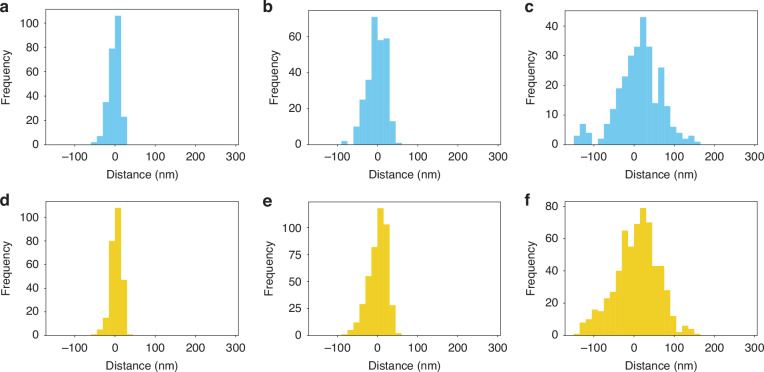
2-color distance analysis between heterochromatin and euchromatin, heterochromatin and functional RNA polymerase for different sizes of heterochromatic clusters. **a–c** 2-color analysis: distance of RNAPII localizations relative to heterochromatin cluster periphery for small (**a**), medium (**b**), and large clusters (**c**), respectively. **d–f** Showcases the 2-color analysis results for H3K27ac localizations relative to heterochromatin cluster periphery for small (**d**), medium (**e**), and large clusters (**f**), respectively

Heterochromatin and euchromatin have different functional associations with the transcription of mRNA. Specifically, the presence of heterochromatin markers such as H3K9me3 within the transcription start sites (TSS) of genes is associated with their repression. Likewise, enhancer-associated euchromatin, which is frequently defined by H3K27ac nucleosome modifications, are modifications to distal elements (segments tens to hundreds of kilobases away from the TSS) that when brought into contact with a gene, promote its transcriptional activity. While it is hypothesized that the functional coupling of heterochromatin, enhancer-associated euchromatin, and active RNA polymerase can be inferred from their spatial relationships, prior to this work, it has not been demonstrated with required resolution to uncover their interactions. Therefore, we utilized our multiplexed SMLM imaging and analysis approach to study the spatial relationship between heterochromatin (H3K9me3) and enhancer-associated euchromatin (H3K27ac), and between heterochromatin (H3K9me3) and RNAPII. After the validation of the algorithms on the simulated dataset, we apply the 2-color distance analysis on H3K9me3, H3K27ac, and RNAPII SMLM data from HeLa cells. Heterochromatin localizations are clustered by DBSCAN with parameters *r* = 50 nm and pts = 3. The heterochromatin cluster’s size is calculated as the polygon size of the cluster’s convex hull. The reported average size of the DNA packing domain is 80 nm in radius^29^. Owing to the distribution in sizes of packing domains, we partitioned heterochromatic clusters based on their effective radii which is defined as the radius of the circle that inscribes the convex hull fit of the cluster datapoints. Clusters were split into small (25–40 nm), medium (40–80 nm), and large domains (80–250 nm) based on their sizes, defined as the radii of the circle with the same size as the convex hull of the clusters. To investigate the locations of surrounding euchromatin and RNAPII, a search radius of 1.5 times the heterochromatic cluster size (radius) is used for distance calculations. The coordinates of each domain’s center are determined by averaging those of convex hull vertices of the cluster (Fig. S5a). To quantify the distance of each euchromatin or RNAPII localization within the searching radius of a heterochromatic domain to the domain periphery, we draw a line connecting the localization point to the domain center (Fig. S5a). The distance from the point where this line intersects the convex hull to the localization point is defined as the distance to the periphery (Fig. S5b, c). If the localization point is outside the heterochromatin domain, the distance to periphery is positive, otherwise it is negative. We found that H3K27ac and RNAPII are generally localized near the heterochromatic domain periphery. In large domains, H3K27ac is closer to the heterochromatic domain center compared to RNAPII, whereas the opposite is observed for medium and small domains (Fig. 3). For large heterochromatin domain: H3K27ac the average distance to the periphery is −1 nm, RNAPII average distance is 8 nm (Fig. 3c, f). For medium heterochromatin domains: H3K27ac average distance is 1 nm, RNAPII distance average distance is −2 nm (Fig. 3b, e). For small heterochromatin domains: H3K27ac average distance is 3 nm, RNAPII average distance is −2 nm (Fig. 3a, d). In conclusion, within the 1.5× searching radius of a H3K9me3 cluster, the average distance of H3K27ac and RNAPII localizations to the periphery is close to zero, indicating a closed structure of heterochromatic domain surrounded by euchromatin and RNAPII. However, the paired distance analysis only elucidates the spatial relationship between euchromatin and heterochromatin or RNAPII and heterochromatin, not providing information on the interaction among all three markers, e.g. where the coupling of euchromatin and RNAPII is regarding the heterochromatic domain.

### Three-color SMLM joint analysis reveals spatial coupling of heterochromatin, euchromatin, and RNA polymerases

**Fig. 4:**
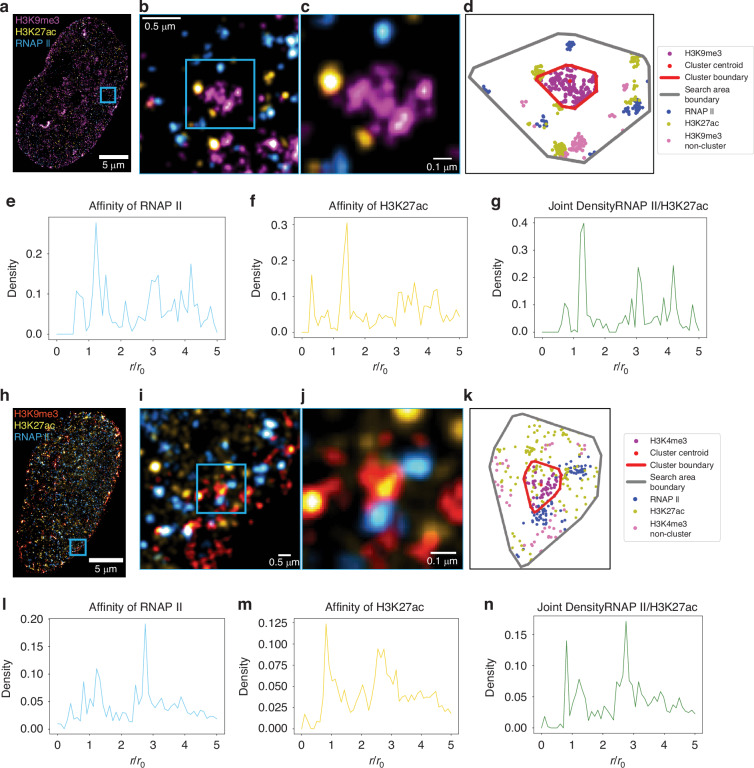
Joint Density Analysis of Multi-label SMLM Imaging in HeLa cells reveals co-existence of RNAPII and H3K27ac on Heterochromatic cluster periphery but overlap in Euchromatic clusters. **a** Shows the three-color SMLM image of a HeLa cell labeled for H3K9me3 (magenta), H3K27ac (yellow), and RNA polymerase II (blue). **b** and **c** Are the highlighted blue insets in the images demonstrating the highlighted heterochromatic cluster with peripheral RNAPII/H3K27ac. **d** Is an example cluster from localization dataset for inset shown in (**c**) plotted. Red Boundary represents the periphery of heterochromatic cluster; Gray boundary represents boundary of analysis region of interest, **e** and **f** Are the joint density of RNA polymerase II to H3K27ac and affinity of H3K27ac to RNA polymerase II regarding H3K9me3 clusters, respectively, across *n* = 4 HeLa cells. **g** Shows the joint density for heterochromatic clusters, combining data from (**e**) and (**f**). **h** Shows three-color SMLM image of a Hela Cell labeled for H3K4me3 (red), H3K27ac (yellow), and RNA polymerase II (blue). **i** and **j** Are the highlighted blue inset demonstrated in (**h**) with (**k**) being the example localizations shown in g plotted with the red boundary being the periphery of H3K4me3 cluster and gray the analysis region of interest boundary. **l** and **m** Are the joint density of RNA polymerase II to H3K27ac and affinity of H3K27ac to RNA polymerase II regarding H3K4me3 clusters, respectively, across *n* = 4 HeLa cells. **n** shows the joint density for euchromatic clusters, combining data from (**l**) and (**m**)

The spatial organization of chromatin and transcription within the nucleus presents a complex landscape of functional interactions. While traditional models posit distinct compartmentalization of heterochromatin, euchromatin, and transcription sites, emerging evidence suggests a more intricate arrangement^27,29,32^. We hypothesized that transcription occurs preferentially at the interface of heterochromatic domains, rather than in spatially segregated regions. To investigate these relationships and compare the organization of heterochromatic and euchromatic regions, we developed a three-color joint analysis method for multi-label single-molecule localization microscopy (SMLM) data. This approach enables the examination of spatial relationships between chromatin components and transcription sites, providing insights beyond those obtainable from conventional two-color analyses. Our approach begins by identifying heterochromatin clusters as seed points. For each cluster, we define a search area extending five times the cluster’s length while maintaining its morphology as defined by convex hull fitting (Fig. S5d). Within this search zone, we locate nearby euchromatin (H3K27ac) and RNA Polymerase II (RNAP II) localizations. We then calculate their distances from the domain center and normalize these distances by the search radius, yielding normalized radial density distributions for both euchromatin and RNAP II. With these points identified, we examine the affinity of each individual-centered target (RNAP II or H3K27ac) for the other. We calculate normalized radial density distributions for two cases: centering RNAP II and searching for H3K27ac (Fig. 4e), and the inverse case of centering H3K27ac and searching for RNAP II (Fig. 4F) within heterochromatic clusters. These distributions are then used to compute the joint density shown in Fig. 4g by taking the geometric mean of both cases (Supplementary Information). To account for localization uncertainty in the SMLM data, we applied this 3-color joint density analysis only to large heterochromatic domains defined in the previous section. We validated our algorithm using simulated data (Fig. 2h), ensuring its reliability for subsequent analyses of spatial coupling between chromatin states and transcription sites.

Using this method, we examined the spatial organization of enhancer-associated chromatin (H3K27ac), active transcription sites (RNAP II), and heterochromatin domains (H3K9me3). Figure 4a–d shows an example HeLa cell labeled with these three markers, visually showing how euchromatin and active RNA polymerases localize around a representative heterochromatic cluster. The normalized radial density distribution of RNAP II localizations proximate to H3K27ac is shown in Fig. 4e, while Fig. 4f presents the inverse case. Figure 4g displays the joint density distribution. Our analysis revealed that the largest peak of the joint frequency distribution for H3K9me3 domains is located outside the normalized cluster size ($\frac{r}{{r}_{0}}=1$), indicating that the most frequent coupling of H3K27ac and RNAP II occur outside the heterochromatic domains. This finding supports the hypothesis that transcription takes place at the interface of heterochromatic clusters.

To compare this organization with euchromatic clusters, we performed a similar analysis using H3K4me3 as a marker for promoter-associated euchromatin. Figure 4h–k shows a HeLa cell labeled with H3K4me3, H3K27ac, and RNAP II. The distribution results for H3K4me3 clusters are presented in Fig. 4l–n. In contrast to the H3K9me3 results, we observed that the coupling of H3K27ac and RNAP II occurs inside the euchromatic (H3K4me3) clusters. These findings challenge the concept of transcription factories and suggest a new model of nuclear organization: heterochromatin domains serve as organizational hubs, with euchromatic regions and active transcription sites preferentially localized at their interfaces. This three-color joint density analysis uncovers a hierarchical chromatin architecture that orchestrates transcriptional activity, providing insights into nuclear function that extend beyond the capabilities of conventional two-color SMLM approaches.

## Discussion

This study introduces the first reported method for three-label single-molecule localization microscopy (SMLM) targeting chromatin in the dense nucleus, achieving uncertainty of ~15–20 nm. Our method enables simultaneous visualization and quantification of multiple chromatin molecular and functional markers, providing insights into the higher-order structure of the genome at the supra-nucleosomal length scale (10–200 nm). This technique, combined with our developed analysis methods, reveals a more nuanced understanding of chromatin organization, challenging previous models and opening new avenues for investigating nuclear architecture and functions. With this approach, we observed cohesive, domain-centered structure rather than spatially distinct groups of chromatin states where heterochromatin domains serve as organizational anchors, with transcriptionally active regions and regulatory elements positioned at their peripheries in a manner that cannot be fully characterized through two-color imaging. The cornerstone of our approach is an optimized sequential labeling protocol, enabling robust labeling of three targets within the dense nuclear environment yielding higher localization density compared to previously published methods^9^ (Fig. S1b). This innovation, combined with our three-label joint density analysis (Fig. S4d–f), allows for the co-registration of three distinct chromatin states or protein localizations, offering a more comprehensive view of nuclear organization than in previous pairwise two-color or diffraction-limited imaging techniques.

Implementation of three-label SMLM within the nucleus required overcoming significant technical challenges unique to the nuclear environment. Unlike the cytoplasm or cellular membrane where multi-label SMLM has been previously applied, the nucleus presents a continuous, densely packed structure where molecular targets exist in close proximity. This dense environment creates substantial challenges for specific labeling, including increased risk of cross-reactivity, reduced antibody accessibility, and potential for non-specific binding as illustrated in Fig. 1a. Our sequential labeling protocol addresses these challenges through carefully optimized blocking steps with species-specific serum and precise incubation conditions. Specifically, we employ modified blocking buffers that include the serum of the host species of our secondary antibodies, used after every target labeling stage. This secondary and tertiary blocking step helps improve target specificity and ensures that all residues for potential off-target binding are blocked before adding our specific primary antibody for the desired targets. Furthermore, overnight incubations at 4 °C are performed to ensure proper labeling. Our protocol is sufficient and optimized for various cell lines used regularly (Figs. 1c and S2), further promoting the ease of implementation for multiplexed labeling of targets within nuclei. This achievement of specific three-color labeling in this environment is particularly significant given that even small increases in non-specific binding or cross-reactivity can compound across sequential labeling steps, potentially obscuring true spatial relationships. Our approach also addresses the limitations of previous chromatin imaging methods. While recent work using structured illumination microscopy (SIM) and lattice light sheet microscopy has provided valuable insights into chromatin organization^10,23,27,43^, these methods are limited by their lateral resolution (>100 nm) and label number. Our approach, with its higher lateral precision of labeled targets and higher spatial frequency of localizations (Fig. S3d), allows for a more detailed examination of chromatin molecular and functional organization at smaller scales with its multi-label capacities. Indeed, we obtain spatial frequencies (~12–15 nm) that align with the Nyquist criteria to probe locations of euchromatin and heterochromatin relative to our anchor heterochromatin clusters. This spatial resolution enables quantitative analysis of the molecular composition and functional organization around packing domains, as the mean distance between localizations for all three labels (~15 nm) is smaller than the length scales of interest (80–200 nm) for examining domain peripheries and interfaces between different chromatin states.

Having established a robust labeling protocol for dense nuclear environments, we next developed analysis methods fully leveraging our multi-label capabilities that were tailored to the complexities of chromatin structure at the packing domain length scale (<100 nm)^29^. Our point-cloud-based method, combined with a Convex Hull fitting algorithm, allows us to base our analysis on actual localizations within each cluster, avoiding bias from assumed shapes. This approach is particularly important given the absence of ground truth morphology for chromatin structures at these length scales. By fitting a polynomial to the cluster periphery based on available points (Fig. S5a), we can more accurately define the boundaries of chromatin domains without imposing assumptions of circular or ellipsoidal shapes (Figs. S6b, S4d). Additionally, our three-color joint density analysis uniquely enables simultaneous measurement of spatial-relationships between the three nuclear targets—a capability impossible with pairwise imaging approaches that only capture binary relationships. This analysis method not only makes use of the three-label dataset but also allows us to determine the distribution of these modifications together. By exploring the affinity of these marks for each other relative to a reference point (Fig. S5d–f), we can quantitatively validate canonical relationships, such as transcription occurring in transcriptionally active chromatin at the supra-nucleosome level, while also elucidating the actual distribution. While two-color imaging can only suggest potential relationships between pairs of marks, three-color SMLM definitively reveals the simultaneous spatial organization of heterochromatin domains, regulatory elements, and transcriptional machinery— relationships that remain hidden in pairwise analysis. Furthermore, our technique allows for the investigation of size-dependent effects on chromatin organization. By partitioning clusters into different size groups, we can observe how the spatial relationships between different chromatin marks change as a function of domain size (Fig. 3). This analysis reveals complexities in chromatin organization that were not apparent in previous studies using lower-resolution techniques or two-color imaging^44^.

Applying these three-label analysis methods to examine the spatial relationship of H3K9me3 (constitutively repressed chromatin), H3K27ac (enhancer-associated chromatin), and RNAPII (active transcription) reveals a cohesive, heterochromatin domain-centered structure. This finding challenges the traditional view of chromatin as being organized into distinct “active” and “inactive” compartments, suggesting instead a more nuanced and interconnected organization. When applied to biological distributions of histone modifications and proteins in HeLa cells, both image-based and point-cloud-based spatial analysis revealed a preference for euchromatic and RNAPII to localize at the periphery of heterochromatin clusters (Figs. 3, 4e–g, and S6d). This observation supports an unprecedented model of functional chromatin organization, where transcriptionally active regions are positioned at the interfaces between different chromatin domains, potentially facilitating rapid transitions between active and inactive states. Interestingly, we found a dependence on cluster size for the resultant distributions. When clusters are partitioned into three groups (Small < 40 nm, Medium 40–80 nm, Large: 80–250 nm), the distributions all center around zero, but from small to large, we observed a shift from a single peak Gaussian-like distribution to a potentially multimodal distribution with a wider range of values (Fig. 3). This size-dependent organization suggests that chromatin domains may have different functional properties depending on their size, with larger domains potentially harboring more complex internal structures. Our three-color joint frequency analysis showed that heterochromatic clusters have a peak joint density on their periphery, while euchromatic clusters have a peak joint frequency within (Fig. 4e–g, l–n). This indicates a joint occupancy distribution rather than a layered one, providing a more nuanced understanding of chromatin organization. The observation that active transcription (RNAPII) and enhancer-associated chromatin (H3K27ac) frequently co-localize at the periphery of heterochromatic domains suggests a model where transcriptionally active regions are positioned to allow rapid access to regulatory elements while maintaining overall chromatin compaction. These findings have important implications for our understanding of gene regulation and nuclear organization. The joint occupancy distribution revealed by our three-color analysis challenges simplistic models of chromatin organization and highlights the importance of considering multiple chromatin states simultaneously. This level of analysis and findings are only possible via the multiplexed labeling and analysis enabled by our protocol, as such this method and pipeline provide a useful tool for probing chromatin compositional organization. These findings, only achievable through simultaneous visualization of three distinct chromatin states in the dense nuclear environment, demonstrate that the nuclear landscape exists as a continuum of intermingled chromatin states rather than discrete compartments, with functional elements like enhancers and active polymerases positioned at the interfaces between different chromatin domains. The ability to simultaneously visualize multiple chromatin states and protein localizations allows for a more comprehensive analysis of how nuclear organization is remodeled during these processes. Future studies using our technique could provide valuable insights into the mechanisms underlying cellular plasticity and the role of chromatin reorganization in disease development.

The integration of our imaging protocol and associated analysis opens exciting avenues for future research. This approach can be extended to study chromatin reorganization during various cellular processes, such as cell differentiation, cell cycle progression, and response to external stimuli. By comparing chromatin structure in different cell types or disease states, we can gain insights into the role of chromatin architecture in maintaining cellular identity and in the development of pathological conditions. Our modified and optimized labeling protocol allows us to simultaneously visualize multiple chromatin marks and protein localizations within the dense nuclear environment and ultimately presents a significant advancement in the field of SMLM for analysis of chromatin and further opens new possibilities for studying the dynamics of gene regulation. Future adaptations of our technique for live-cell imaging could provide unprecedented insights into how chromatin organization changes in real time during processes such as transcription activation or cellular differentiation. Combining multi-color SMLM imaging and advanced spatial analysis algorithms enables the extraction of meaningful information from complex biological datasets and generates testable hypotheses for future studies. Our work not only enhances our understanding of chromatin architecture but also provides a robust methodology for future investigations into the interplay between chromatin structure, gene transcription, and epigenetic processes.

## Materials and methods

### Cell culture

HeLa cells were grown in RPMI 1640 medium (Thermo Fisher Scientific, Waltham, MA; catalog number 11875127). The medium was enriched with 10% fetal bovine serum (Thermo Fisher Scientific, Waltham, MA; catalog number 16000044) and penicillin-streptomycin (100 μg/ml; Thermo Fisher Scientific, Waltham, MA; catalog number 15140122).

### Imaging buffer preparations

DABCO [1,4-Diazabicyclo-(2.2.2)-octane] (D27802, Sigma) was dissolved in distilled water to prepare a 1 M stock solution, with 12 M HCl added until the powder was completely dissolved, and the pH reached 8.0. Maintaining this pH is crucial as it primarily determines the pH of the overall buffer. The stock solution can be stored in the refrigerator, in the dark, for several weeks. DTT 1 M (43816, Sigma) was used directly as purchased and stored in the refrigerator for several weeks. Sodium sulfite (S0505, Sigma) was dissolved in 10× PBS to a concentration of 1 M and can be kept at room temperature on the bench for several weeks. Typically, we prepared 40 mL of buffer at a time, adjusting the pH with NaOH and HCl using a pH meter, and then stored it in the refrigerator.

### 3-color SMLM Sample preparation

Primary antibodies rabbit anti-H3K9me3 (Abcam), mouse anti-H3K27ac (Thermo-Fisher), rat anti-RNA Polymerase II (Abcam), and mouse anti-H3K27me3 (Abcam) were aliquoted and stored at –20 °C. Secondary antibodies goat anti-rabbit AF647 (Thermo-Fisher), goat anti-rabbit AF568 (Thermo-Fisher), goat anti-rat AF488 (Thermo-Fisher) were stored at 4 °C. For catalog numbers and buffer conditions see supplementary information.

The 3-color SMLM sample preparation has three sequential staining processes for three targets.

1. The cells were plated on No. 1 borosilicate bottom eight-well Lab-Tek Chambered cover glass with 12.5k cells in each well. After 48 h, the cells were fixed in 3% paraformaldehyde and 0.1% glutaraldehyde in PBS for 10 min, and then washed with PBS once, quenched with freshly prepared 0.1% sodium borohydride in PBS for 7 min, and rinsed with PBS three times at room temperature.
2. The fixed samples were permeabilized with a blocking buffer (3% bovine serum albumin (BSA), 0.5% Triton X-100 in PBS) for 20 min
3. Samples were incubated with rabbit anti-H3K9me3 (Abcam) in a blocking buffer for a minimum of 2 h at room temperature and rinsed with a washing buffer (0.2% BSA, 0.1% Triton X-100 in PBS) three times.
4. The fixed samples were further incubated with the corresponding goat secondary antibody–dye conjugates, anti-rabbit AF647 (Thermo Fisher), for 40 min, washed thoroughly with PBS three times at room temperature and stored at 4 °C. Upon this step, the staining for the first target (H3K9me3) is finished.
5. Sample is then incubated overnight in a modified blocking buffer with goat serum for the following 2-color and 3-color staining (see Supplementary Information).
6. After the overnight blocking step, the sample would go through the same process as Steps 2 and 3, however, with modified blocking and washing buffers (see Supplementary Information). The second target primary antibody, in this case is a rat anti-RNA Polymerase II (Abcam) and the secondary antibody is goat anti-rat AF488 (Abcam).
7. Repeat steps 5 and 6 for the third target (overnight blocking, then primary and secondary antibody incubations) for the third target with primary antibody of mouse anti-H3K27ac (Thermo Fisher) and the secondary antibody is goat anti-rat AF568 (Abcam). Once the secondary antibody step is done, wash two times in PBS and store samples at 4 °C if not imaging immediately.

### SMLM system configuration and imaging

The STORM optical instrument was built on a commercial inverted microscope base (Eclipse Ti-E with the perfect focus system, Nikon). Two continuous lasers were used for illumination, one of which has five different wavelengths (405, 488, 532, 552, and 637 nm, OBIS Laser Box, Coherent) while the other is a green laser with 532 nm emission (MGL-FN-532, Changchun New Industries Optoelectronics Tech. Co., Ltd.). The lasers are collimated with an average power at the sample of 3–10 kW/cm^3^. Images were collected via a ×100 1.49 numerical aperture (NA) objective (SR APO TIRF, Nikon) and captured on an electron-multiplying CCD camera (iXon Ultra 888, Andor). At least 10,000 frames with a 30 ms acquisition time were collected from each sample for each wavelength channel. All images were acquired using a total internal reflectance fluorescence illumination (TIRF) schema which allowed for evanescent wave illumination of ~200 nm slabs from the glass cell interface. Our focal plane was set within this depth to capture properly excited in-focus fluorophores. The labeled samples—H3K9me3 or H3K4me3 (AF647), H3K27ac (AF568), and RNA Polymerase II (AF488)—were imaged sequentially using a 647 nm laser first, followed by a 532 nm laser, and finally, a 488 nm laser to minimize inter-channel photobleaching from shorter-wavelength light.

## Supplementary information

Supplementary Information with Figures

## Data Availability

The data and analysis codes that support the findings of this study are available from the corresponding author upon reasonable request. The raw image data used for this manuscript will be deposited in the Dryad Digital Repository (DOI to be generated upon acceptance) and will be made publicly available upon publication.
