# Supplementary material for: Three-color single-molecule localization microscopy in chromatin
Source: Light Sci Appl. 2025 Mar 17;14:123. doi: 10.1038/s41377-025-01786-1 (PMC11911409; doi:10.1038/s41377-025-01786-1)
Supplement: Supplementary file 1 — Supplementary Information with Figures [file 41377_2025_1786_MOESM1_ESM.pdf]

SUPPLEMENTARY INFORMATION FOR: Three-color single-molecule localization microscopy in chromatin

Nicolas Acosta[1,2]†, Ruyi Gong[1,2]†, Yuanzhe Su[1,2], Jane Frederick[1,2], Karla I. Medina[1,2,4], Wing Shun Li[1,2,5], Kiana Mohammadian [1], Luay Almassalha[1,2,3], Geng Wang[1,2*], Vadim Backman[1,2*]

[1.] Department of Biomedical Engineering, Northwestern University, Evanston, IL 60208, USA.
[2.] Center for Physical Genomics and Engineering, Northwestern University, Evanston, IL 60208, USA
[3.] Department of Gastroenterology and Hepatology, Northwestern Memorial Hospital, Chicago, IL 60611, USA
[4] IBIS Interdisciplinary Biological Sciences Graduate Program, Northwestern University, Evanston, Illinois, 60208, USA

[5] Applied Physics Program, Northwestern University, Evanston, Illinois, 60208, USA

† These authors contributed equally to this work.
*Corresponding author. Emails: wanggeng@northwestern.edu, v-backman@northwestern.edu

SUPPLEMENTARY FIGURES, TITLES AND LEGENDS

Supplementary Figure 1

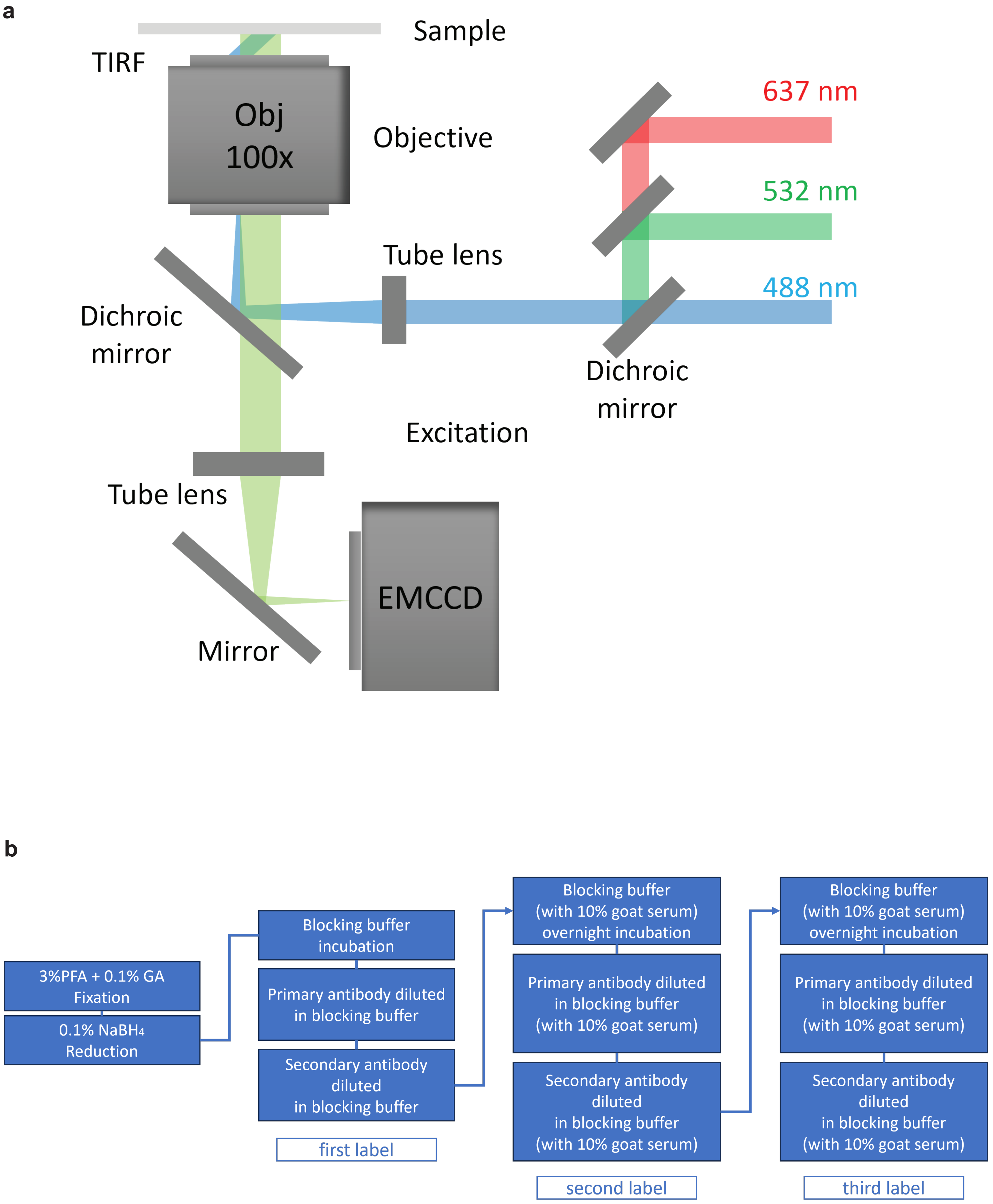

Supplementary Figure 2

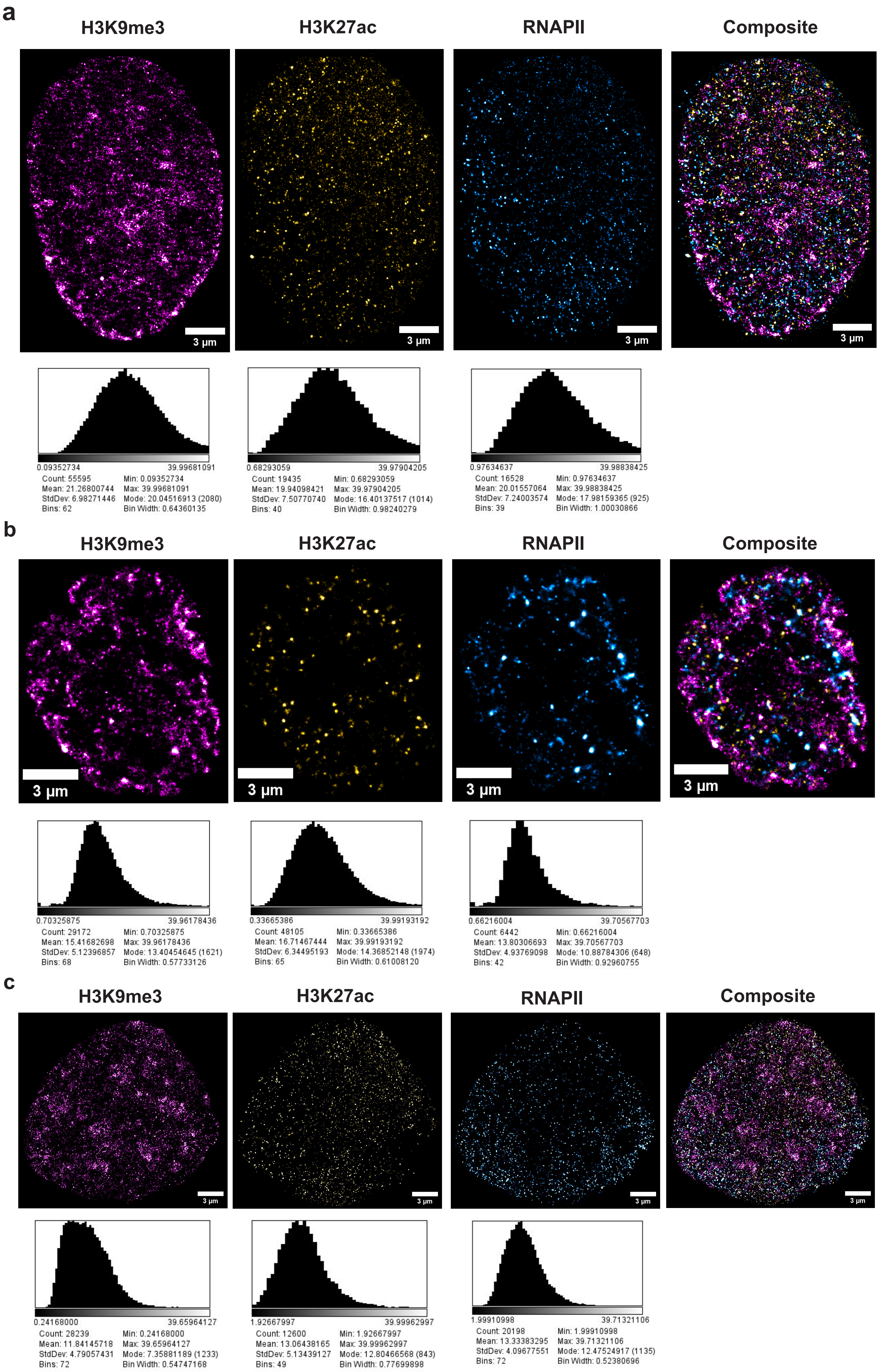

Supplementary Figure 3

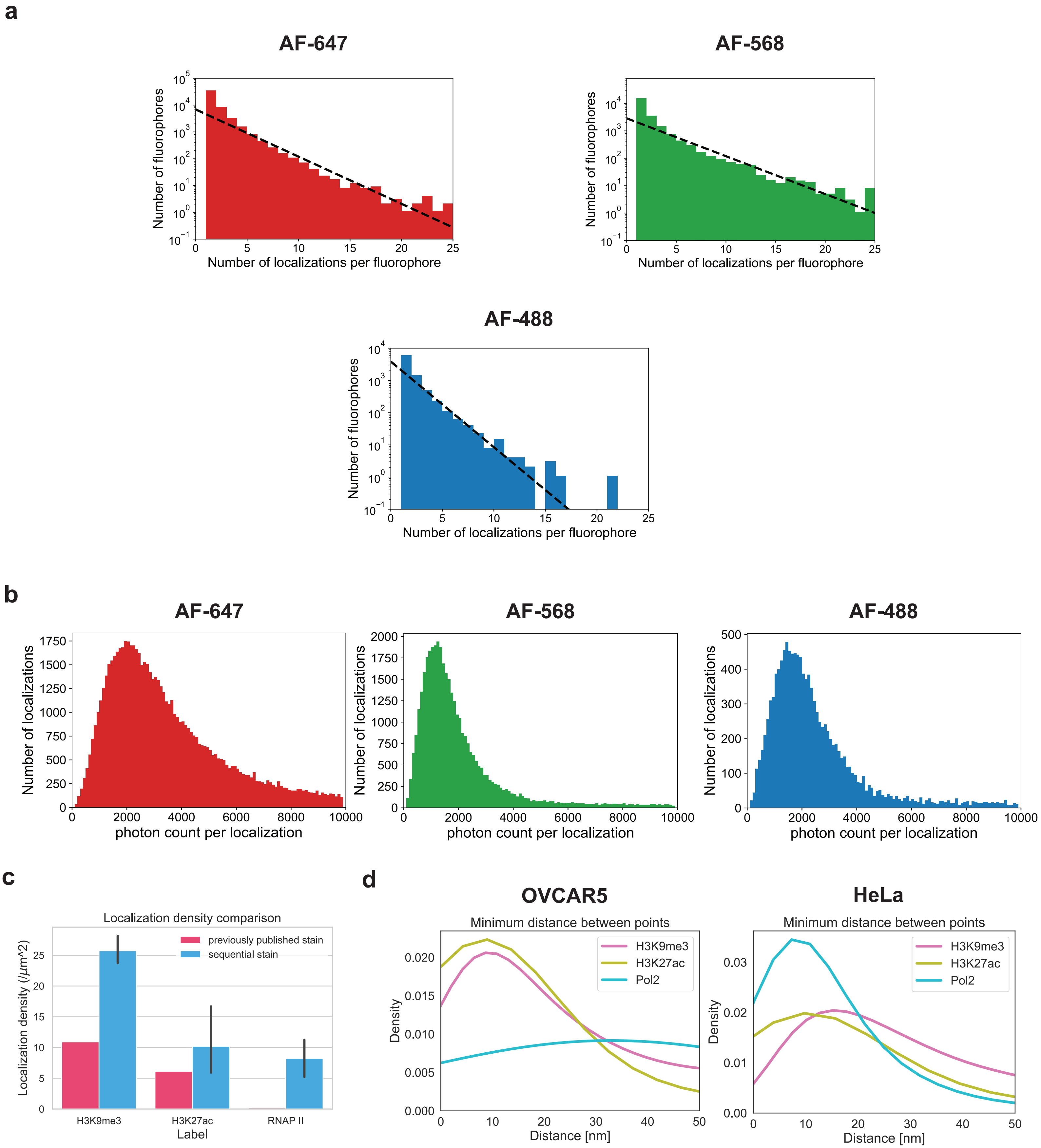

Supplementary Figure 4

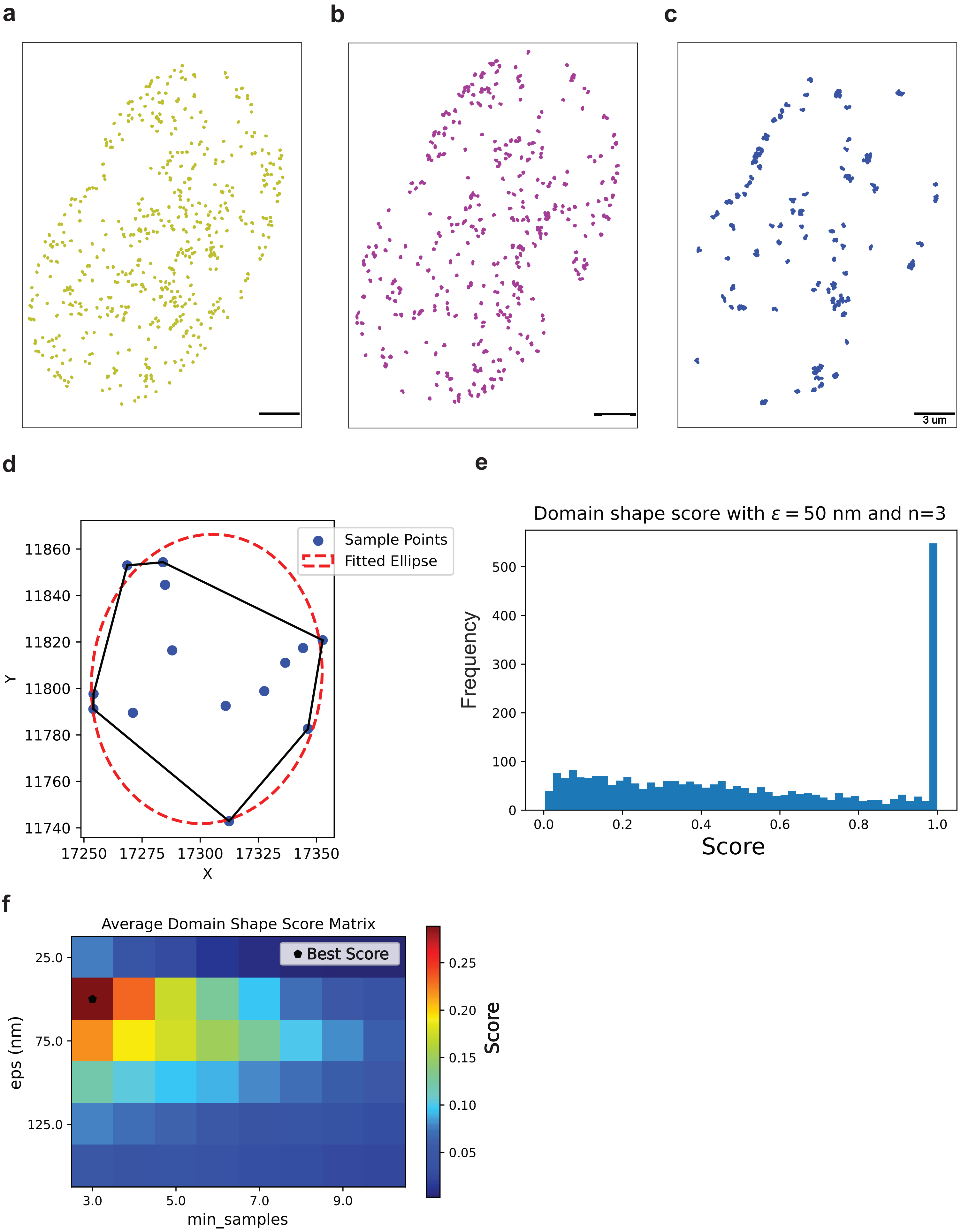

Supplementary Figure 5

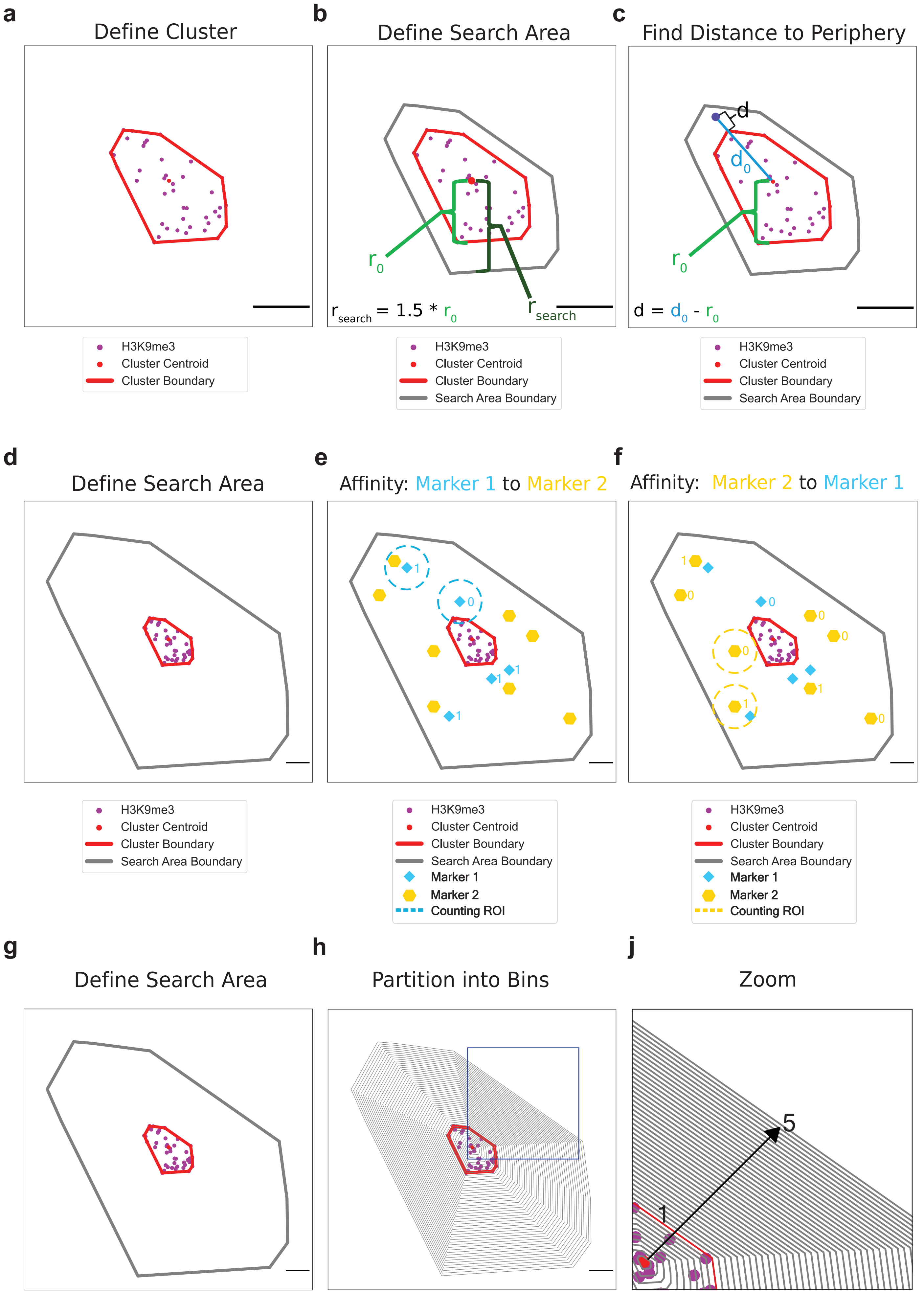

Supplementary Figure 6

a

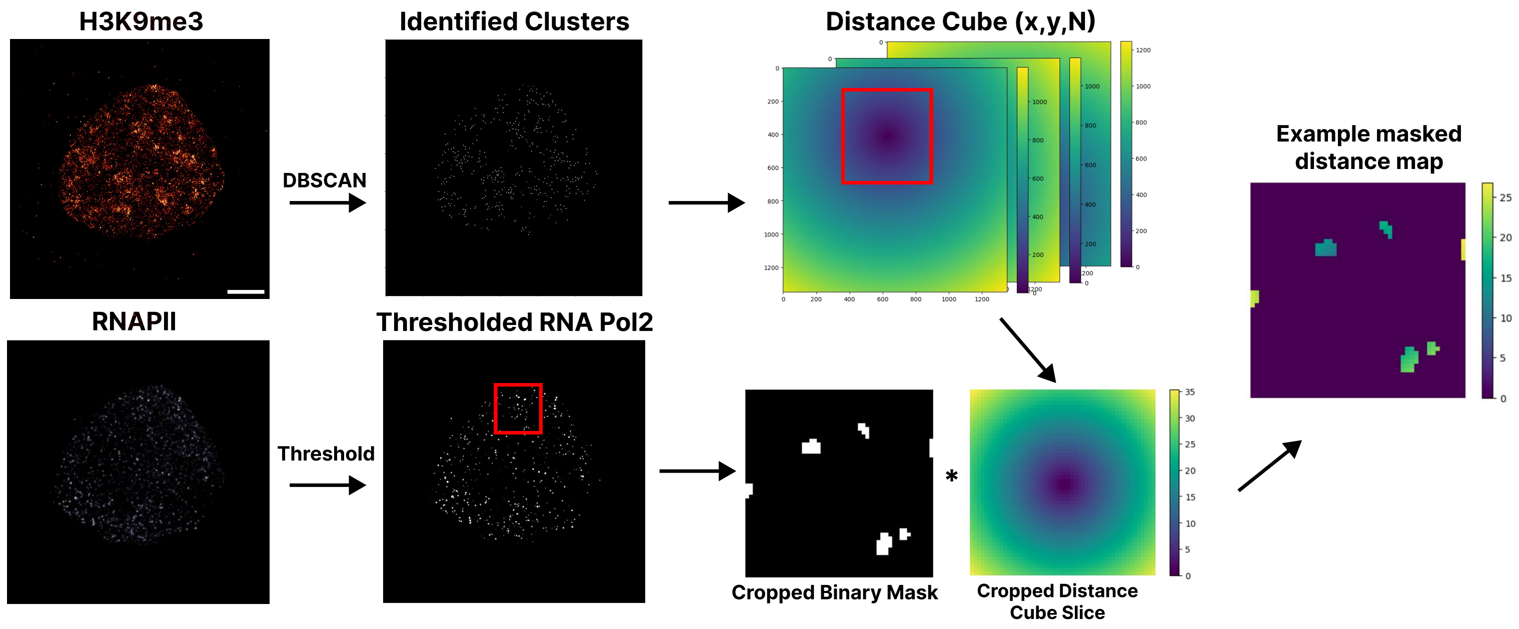

b

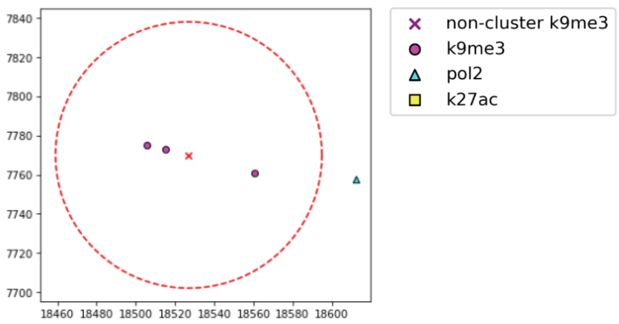

Supplementary Figure 7

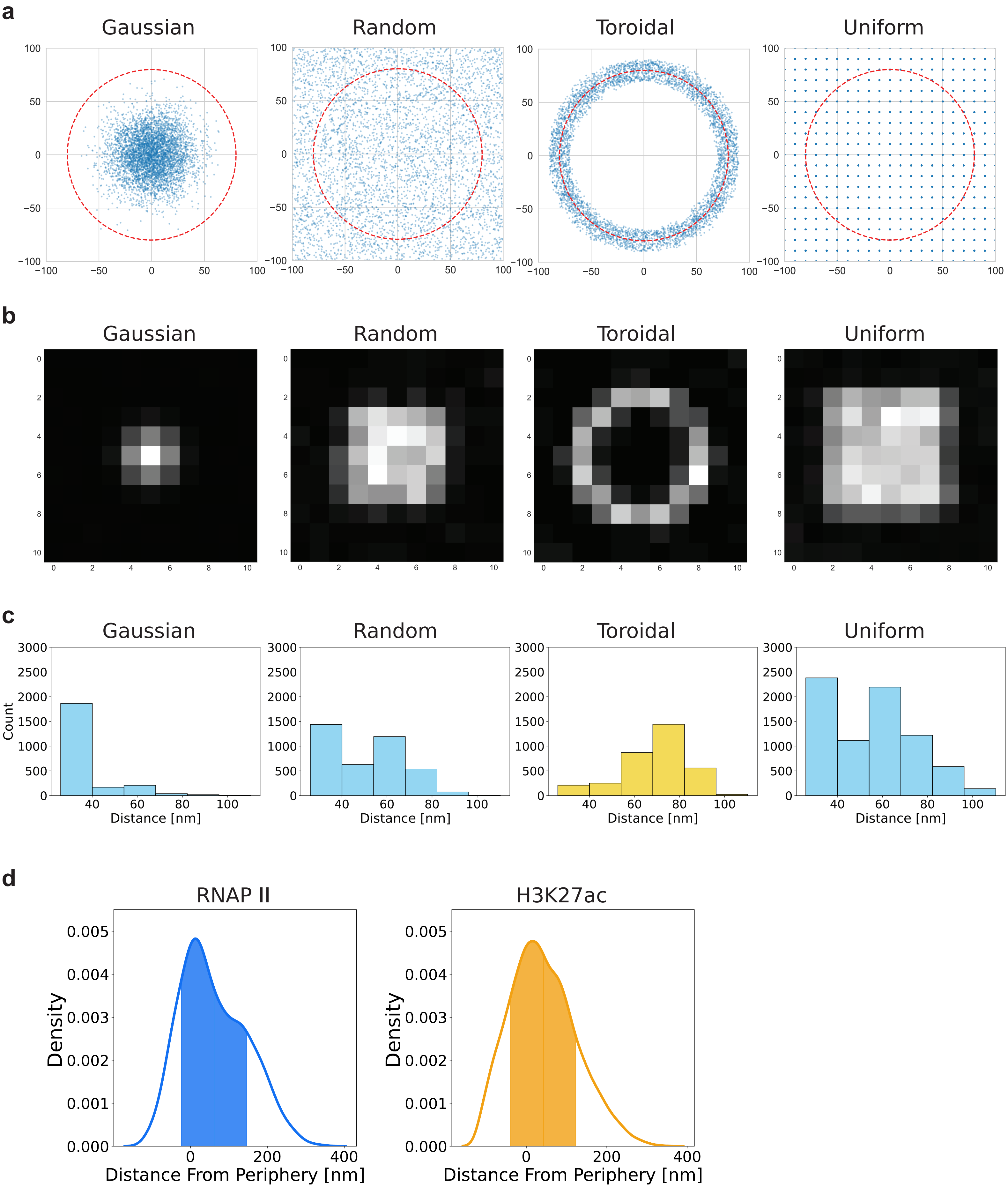

Figure S1. Schematic of three-color single-molecule localization microscopy and labeling protocol schematic

(a) All the data were collected on this Eclipse Ti-E NIKON microscope. 3 labels (AF647, AF568 and AF488) were excited by laser line 637 nm, 532nm, 488 nm respectively. **(b)** describes the sequential staining protocol for 3-color multiplexed single molecule localization microscopy sample preparation.

Figure S2. Example three-color SMLM images from BJ Fibroblast and HCT116 showcase functionality of labeling protocol in various cell lines

(a) Showcases individual channels (H3K9me3: magenta, H3K27ac: yellow, and RNAPII: cyan) and composite image of all three channels in BJ fibroblasts. **(b)** Showcases the same as in A but in HCT116 cells. **(c)** Showcases the same as in A but in HCT116 cells. All scale bars are 3 μm. Each image has the uncertainty in localization (localization precision histogram) obtained post ThunderSTORM below for reference.

Figure S3. Multi-blinking Count, Localization Density and Spatial Frequency comparisons for multi-label SMLM protocol and imaging.

(a) Plots the histogram showing the number of fluorophores that blink *n* times. This is plotted for all secondary antibodies used in this manuscript (AF647, AF568 and AF488). All distributions have most fluorophores blinking less than 2 times during acquisition. Average *n*: 1.639 (AF647), 1.848 (AF568), 1.553 (AF488). **(b)** Semi-log version of plots in (a). The coefficients of determination R^2 of the linear regression lines for AF-647, AF568 and AF488 are 0.92, 0.90, 0.96, indicating that the histograms in (a) are exponential decays. **(c)** Photon count histogram for secondary antibodies used in this manuscript. The results in (a) (b) were calculated from the spectroscopic single molecule localization microscopy data of the MCF10A cells. Since the blink properties mostly depend on the fluorophore type and the buffer, as well as the illumination density. The result here can also reflect the characteristics of the same three types of the fluorophores used in the HeLa cell data analyzed in Fig. 3, 4. **(d)** Localization density for each labeling protocol. We observe a higher localization density for our sequential labeling protocol (HeLa *n*=4) than in the combined labeling case (OVCAR5 *n*=1). **(e)** Minimum distance (spatial frequency) between localizations KDE for each targeted structure (H3K9me3 – AF647, H3K27ac – AF568, and RNAPII – AF488) for poor quality image (OVCAR5, simultaneous staining) vs good quality image (HeLa, sequential staining). Density curves showcase low median spatial frequencies (~15-20) for all three labels with sequential labeling, whereas simultaneous labeling protocol does not produce consistent spatial frequency for all three labels. It provides a potential metric for assessing the quality of labeling because extreme sparse localizations will yield high minimum distance between localizations.

Figure S4. Domain Shape Score parameter search method and Example partitions of DBSCAN identified heterochromatic clusters

(a-c) Identified heterochromatin (H3K9me3) clusters from DBSCAN with epsilon = 50 and minimum number of samples = 3 for (small <40 nm), medium (40-80 nm) and large (80-250 nm) clusters respectively. **(d)** Example ellipse fitting method used in Domain Shape Score algorithm. Sample points with fitted periphery via Convex Hull method in black with fitted ellipse in dashed red. **(e)** Histogram of Domain Shape Score values for all identified clusters in a single nucleus example for epsilon =50 and minimum number of samples = 3. **(f)** Parameter grid search for using Domain Shape Score as optimizing parameter. (50,3) provided the highest score for

heterochromatin (H3K9me3) dataset indicating proper parameters for subsequent cluster analysis.

Figure S5: Two Marker analysis and Three marker analysis diagrams demonstrating counting strategy for multi-label SMLM analysis algorithms.

(a-c) Pictorially demonstrate the steps for two color SMLM analysis. The first panel demonstrates periphery fitting of DBSCAN identified cluster. B demonstrates the analysis region definition which entails a 1.5 * r0. (c) demonstrates distance calculation which measures distance in the direction of mark relative to the centroid of a cluster but subtracts the radial distance of the vector from center to periphery boundary. Only remaining distance measured relative to periphery with directionality considered. **(d-f)** Pictorially demonstrates the steps for three color SMLM counting. (d) is the same as (b) however the analysis region for three color SMLM is 5 times the radius of the cluster. (e) Is the affinity of Marker 2 (yellow) to Marker 1(Cyan). This is done by counting the number of times there is a marker 2 present within 100 nm of marker 1. (f) is the same as (e) but instead of centering marker 1, marker 2 is centered and we count affinity of marker 1. This counting method is used to generate the joint density affinity plots shown in Fig. 4. **(g-h)** demonstrate how the area for 3 color analysis is partitioned for Joint Density plots. (H) Demonstrates that partitioning into 50 bins such that every step of r0 (1*r0, 2*r0…) has 10 bins between. (j) is a zoom showing the partition. 1 delineates the red periphery line of the cluster and 5 is the outer most boundary.

Figure S6: Proposed Analysis pipeline for Image based approach for multi-label SMLM

(a) demonstrates the analysis for 2-color analysis for H3K9me3 and RNAPII. The premise of this approach is to use H3K9me3 as seed points for analysis and uses the centroid of the identified DBSCAN clusters for analysis reference points. Subsequently, a distance cube, where each slice corresponds to an identified cluster is used to calculate all possible distances relative to the heterochromatic cluster. We use a binarized image of the second target. In this case the RNAP II, to mask the distance cube for a given cluster and show the remaining distance pixels. Pixels across all clusters are concatenated and then used as dataset for analysis for a given mark. The goal is to understand the spatial distribution of a given mark relative to seed reference point. **(b)** showcases example structure when assuming a circle with radius half the largest distance between the points that compose the structure.

Figures S7: Simulated distributions and their image counterparts as inputs for Image based 2-label SMLM analysis demonstrate algorithm robustness.

(a) Scatter plot of simulated distributions for Gaussian, Random, Toroidal and Uniform test cases. Distributions shown are the sum of all sampled points such that the resultant distribution across all clusters follows a given spatial distribution. **(b)** Image based counterparts for simulations shown in (**a**). Pixel size in the image is 26 nm. Similar to (a) the image represents the sum of all samples that were deposited around cluster seed points to have trend that follows the given spatial distribution. **(c)** Count Histogram results for spatial Euclidean distance measurements of given distribution relative to heterochromatin cluster center. Gaussian, Random Toroidal and Uniform test cases resulted in visually distinct distributions. **(d)** Kernel Density estimations for

distance distributions of RNAP II and H3K27ac relative to H3K9me3 clusters in HeLa Cells (n=4, clusters = 5000) using same image-based method describe in Fig. S5. Distance is measured relative to the periphery and shows similar centering near the periphery of these heterochromatic clusters.

SUPPLEMENTARY METHODS AND MATERIALS:

Data acquisition, pre-processing and localization estimate parameters:

Using the system configuration and imaging parameters described in the methods section, we iteratively acquired images for each channel of interest (red: 637 nm, green: 532 nm, blue: 488 nm) corresponding to our label of interest. Each image stack acquired at least 10,000 frames at 30 ms exposure such that at the end of our acquisition we had over 30,000 frames across all three channels. Lateral Positions (x,y) for our stage were acquired at the beginning of each channel's acquisition to account for any lateral drift during the collection. Pre-processing of our data was done via an ImageJ macro that was made to automatically apply background subtraction steps. The first step is an image subtraction of the minimum projection of each channel's stack from each frame. Thereafter we use the built-in rolling ball background subtraction tool with a rolling ball radius of 5 pixels for all images in the stack. This is done for each channel acquisition and then all resultant pre-processed stacks are analyzed with the built-in Thunder-STORM Image J plugin. The camera setup for this plug-in is dependent on your imaging configuration, in our case we used a pixel size of 130 nm (camera pixel pitch/objective magnification), Photoelectrons per A/D count: 18.6, Base level [A/D counts]: 0, EM gain: 240. Peak intensity threshold coefficient was set dependent on acquisition quality but ranged from 2-3. Localization estimates were performed using the maximum likelihood estimation assuming a gaussian PSF with a fitting radius [pixel] of 4 and an initial sigma [pixel] of 2.5. Image reconstructions were generated using the average shifted histogram algorithm with a magnification of 5 and update frequency of 50 frames. Once data was generated, we applied the built in cross-correlation drift correction and a filtering of datapoints to omit localization with uncertainty larger than 40 nm. Once done, these estimation datasets were saved as csv format and input into our python clustering framework and analysis pipeline.

Multi-blink counting and minimum distance

In Fig. S3a, we calculated the number of localizations per fluorophore detected based on the spatial and spectral similarity in a restricted moving time window. The algorithm is adapted from Dong et al, using a spectroscopic SMLM system. Firstly, we set the maximum spatial difference as 2 times of the average uncertainty and maximum spectral centroid difference as 5 nm for localizations characterized as the emissions from the same fluorophore. Then we applied a temporal restriction: If two temporally adjacent localizations, identified as originating from the same fluorophore based on the above metrics, exhibited a temporal difference exceeding 200 seconds, they would be considered as emissions from distinct fluorophores. The results showed the average

localizations per fluorophore for AF647, AF568, AF488 are 1.639, 1.848, 1.553 respectively. This suggests that the 3-color SMLM image can be interpreted as an approximate representation of density maps, supporting the validity of the use of DBSCAN clustering algorithm. In Fig. S3c, we investigated the minimum distance between any localization to all other localizations in the samples gone through the simultaneous staining method compared to those stained by our sequential staining method.

Algorithm Descriptions and Considerations:

The motivation and explanation of each algorithm is detailed below. For a pictorial depiction of each algorithm pipeline see Fig. S5.

Image Based Analysis Algorithm: Image based analysis algorithm, or the hybrid analysis algorithm, (Fig. S5a) relies on the DBSCAN method from the scikit-learn Python library to cluster the heterochromatic marks. Circles are used to describe the geometrical properties of each cluster due to its simplicity and we acknowledge that this assumption creates a limitation in identifying the actual shape of a cluster. For each cluster, the center of the circle is chosen to be the geometric centroid of the cluster points, and its effective diameter is defined by the maximum Euclidean distance between all point pairs. Yet since the cluster points fall within the pixel size of our reconstructed images (26 nm), a image based segmentation of the border of our heterochromatic clusters is not possible, thus this approach was taken. After determining the clusters, their centroids and their boundaries, we create distance cube (X, Y, N) where X, Y are the dimensions of the reconstructed image as defined as 5 times the size of the size of our ROI during acquisition, and N was the number of clusters identified after size filtering. Filtering for size was done to ensure no clusters that were beneath our precision or too large to be biologically relevant were considered in the analysis. These thresholds are the same as mentioned in the main text. Each slice in the distance cube corresponds to each cluster such that slice indexing (0,1,2, 3,…,N) correspond to clusters indexing (0,1,2,3,…,N). This was done to easily keep track of each cluster in visual format. This distance cube, as shown in Fig. S3a, has values of each pixel relative to the centroid of that given cluster. For example, the first slice is based on the cluster (index 0) has distance values corresponding to its centroid (x0, y0) and the next slice which has a different cluster centroid (index 1) will base its distance values of (x1, y1). The logic behind this was to calculate all distance values once and not repeatedly calculate distance each time for each cluster. With a distance cube that contains the respective pixel distances to each identified cluster, we then used the reconstructed images for the other two labeled targets, in this case RNAPII and H3K27ac, and used binary thresholding to keep pixels with values greater than 250. Using a set threshold was done to ensure only the brightest pixels since it is assumed that they have similar target values. This selection would allow us to count high occurrences of our labeled targets in our distribution analysis. We use the average shifted histogram algorithm that is pre-built into the ThunderSTORM Image J plugin to reconstruct our images. Since this is essentially binning the localizations into respective

bins, the brighter the pixels indicate the more estimated localization datapoints in that bin, as such we want to use a set threshold for our spatial distance analysis. This ensures that we consider a similar number of events when measuring distance relative to our clusters. With binarized images, we then mask the distance cubes such that all remaining pixels in the distance cube slice would correspond to enriched areas of our markers. The remaining pixels are then recorded and then adjusted either to report distance to centroid (no change) or distance to periphery (distance – cluster size). All distances were concatenated into one array across all imaged cells included in analysis and then processed to generate distance histograms and kernel density estimation of distance to arrive at the distribution of distance of our markers (RNAPII and H3K27ac) relative to our heterochromatic clusters.

Point Cloud Analysis Algorithm: Paired Distance and Joint Density measurements: This analysis method uses the points identified via Thunder-STORM directly and as such is referred to as point-cloud, since the scatter of points often resembles a cloud of particles. Point-cloud algorithms used in this study started with the same DBSCAN clustering method but differ on use of clustered data. Cluster boundary was defined either by fitting using the scikit-learn Convex Hull method to fit a polynomial to the external cluster points. This approach was taken as it based our reference point, the periphery, on data that was acquired and made no assumptions on the morphology which was a limitation of the previously described image-based approach. This cluster boundary is the reference point for both the Euclidean distance measurements to the other targets (2-color paired analysis) and the Joint density analysis (3-color analysis). To start, the identified periphery was used to define an analysis region of interest by doing a 1.5 times scaling of the original vertices of the convex hull defined cluster periphery. Please see the following section for the justification of this scaling value and its use in our analysis. Distance to our labeled targets, RNAP II and H3K27ac, was measured relative to the periphery by drawing a vector from the centroid of the cluster to the localization (RNAPII/H3K27ac) in question. The distance from the centroid to the cluster boundary is then measured along that same vector and the difference of these measurements is assigned to that specific localization. This is done for all labeled targets within the analysis window and then for all clusters. Again, the clusters used in analysis here were filtered for size as described in the main text. The distance results are then visualized in a standard histogram as shown in Fig.3 b.

For three label analysis, an analysis window of 5× scaling of the area of the original cluster is used. Prior to calculating joint density, the individual co-inhabitance of the marks is measured by going to every localization of either marker and counting the occurrences of the other marker not centered within a 100 nm radius circle (Fig.S5). If there is co-inhabitance of the centered mark and the other labeled target, we then assign a numerical value of 1, if there is not then that localization remains at a base value of zero (Fig. S5). These values are then assigned a distance relative to the original cluster size r0, such that they fit into bins which are expansions upon the original cluster. 50 expansions are used for each cluster, and with normalization of distance to the original cluster size, the co-inhabitance data points are then placed into bins from 0-50 (r/r0). Normalization allows

for concatenation of co-inhabitance for a given pairing (RNAPII in H3k27ac and vice versa) since they are all normalized to the cluster size r0. Results for each cluster are combined to give a plot for affinity of one target to the other (see Fig. 4) and then the joint density is given as the combination of each individual plots by taking the counts for each bin and calculating the geometric mean. This is done for each bin from 0 to 5 times the original area. Once this is done, we have the joint density plots as seen in Fig. 4. These plots are useful due to their ability to highlight enriched areas of both marks to give a better understanding of the distribution of these labeled targets.

Simulated Distributions Generation:
To test the robustness of the algorithms, we generated sample distributions that were spatially distinct. These sample distributions were tested on both image and point-cloud based Euclidean distance measurements as well as on the 3-color point-cloud joint density algorithm. To generate these samples, we sampled according to the distribution requirements. Sampling was done such that the average distribution around all clusters would follow the distribution of interest. The number of samples to be deposited was calculated by finding the average density of the given mark (RNAP II/H3K27ac) in a circular area around the cluster defined by $r = r_0\sqrt{2}$ such that the area of the annulus outside the cluster is equal to that inside the cluster with assumed radius r_0. The number of simulated marks deposited would then be given by $n = density_{mark} * 2\pi r_0^2$. This sampling is relevant for all distributions other than unform where the samples are deposited at uniform positions of 10 nm around the cluster. Samples were deposited around each analysis area around each cluster and representative images of the scatter plots of the resultant distributions for all clusters are shown in Fig. S6a. For image-based algorithm, the samples were binned to the nearest 26 nm pixel and deposited around each identified cluster. Resultant overlays of all samples on one image are shown in Fig. 6b.

DBSCAN parameter grid search algorithm:
Appropriate clustering parameters, epsilon and number of points per cluster, were evaluated by Domain Shape score which considers the size and shape of clusters in an equal manner. We developed this score and performed a parameter grid search to find the optimal epsilon and number of points to accurately capture our targeted structures.

The premise of our scoring involves fitting of an ellipse to the DBSCAN identified clusters and then seeing which parameters optimize our fitting. The optimization is based on a scoring function shown below as well as lower and upper bounds of size which follow biologically relevant sizes identified previously in a publication via Chromatin Scanning Transmission Electron Microscopy[26].

The general scoring function follows:

$$P = (1 - \varepsilon') \cdot f(S) \quad (1)$$

Where Epsilon is the eccentricity of an ellipse, and f(S) is a size function that considers the size of an ellipse ($S = \pi ab$) and has the form of:

$$f(S) = \begin{cases} \frac{S}{\pi r_{lb}^2} & if\ S < \pi < \pi r_{lb}^2 \\ 1 & if\ \pi r_{lb}^2 \leq S \leq \pi r_{ub}^2 \\ \frac{\pi r_{ub}^2}{S} & if\ S > \ \pi r_{ub}^2 \end{cases} \quad (2)$$

Equation 2 is used to calculate score with equation 1 for all domains. Once that is done, we do a weighted average with weight proportional to size S. The weighted average will further punish the scores and follows the following form:

$$P = \varphi * \sum_{i=1}^{n} \omega_i p_{pi} \quad (3)$$

Where φ is the usage efficiency that considers the total number of points being used to classify clusters out of all localization points in the dataset. This is used to penalize parameter sets that lead to very large clusters or those that discard many of the clusters as noise. This weighted average will be done for every domain from i=1 to n and will weigh the scores calculated in equation one by their respective weight.

Ellipses were fit where at least three or more peripheral cluster points were considered for circle fitting and five or more points were considered. Normalization of area was done via dividing estimated cluster area by area of a circle of with radius 80 nm. This approach is based on SMLM clustering, however future directions should consider using a ground truth dataset coming from Electron Microscopy or another technique that can resolve domains directly. In this manner, the domain shape score will aim to establish clustering parameters that recapitulate the known size and locations of clusters that have been established *a priori*. Results of grid search can be found in Fig. S3.

Our basis for these analyses is the heterochromatic cluster, which we establish with the standardized DBSCAN algorithm and inform our cluster threshold for size based on prior published identification of chromatin packing domains via electron microscopy[51]. Selecting appropriate parameters for clustering in SMLM data is crucial, as incorrect choices can lead to misleading results. If the epsilon value or the number of points is too high, large chromatin clusters may form, obscuring sub-diffraction chromatin aggregations; too low, and clusters of interest may fragment into smaller, insignificant groups. This parameter optimization is a critical first step, as varying target densities necessitate tailored settings to avoid biasing downstream analyses, especially those relying on cluster peripheries for distance and joint inhabitance studies. While DBSCAN is a useful density-based clustering algorithm, it requires fine-tuning to accurately identify clusters that reflect the biological structure's size.

Reagent List:

**Reagent**	**Components**
Primary Antibodies	H3K9me3 (ab176916, Abcam)<br>H3K27ac (MA5-23516, Thermo Fisher)<br>H3K4me3 (ab213224, Abcam)<br>RNAPII (ab252855, Abcam)

Secondary Antibodies	Goat anti Rabbit AF647(A21245, Thermo Fisher)<br>Goat anti Mouse AF568(A11004, Thermo Fisher)<br>Goat anti Rat AF488 (A150157, Thermo Fisher)
Blocking Buffer	3% Bovine Serum Albumin (BSA)<br>0.5% Triton X-100<br>In 1xPBS<br>Modified:<br>Same as Above + 10% Goat Serum
Washing Buffer	0.2% Bovine Serum Albumin (BSA)<br>0.1% Triton X-100<br>In 1X PBS<br>Modified:<br>Same as Above + 1% Goat Serum

Detailed Protocol:

The 3-color SMLM sample preparation has 3 sequential staining processes for 3 targets.

1. The cells were plated on No. 1 borosilicate bottom eight-well Lab-Tek Chambered cover glass with seeding density of 12.5k. After 48 h, the cells were fixed in 3% paraformaldehyde and 0.1% glutaraldehyde in PBS for 10 min, and then washed with PBS once, quenched with freshly prepared 0.1% sodium borohydride in PBS for 7 min, and rinsed with PBS three times at room temperature.
2. The fixed samples were permeabilized with a blocking buffer (3% bovine serum albumin (BSA), 0.5% Triton X-100 in PBS) for 20 min and then incubated with rabbit anti-H3K9me3 (Abcam) in blocking buffer for a minimum of 2 hours at room temperature and rinsed with a washing buffer (0.2% BSA, 0.1% Triton X-100 in PBS) three times.
3. The fixed samples were further incubated with the corresponding goat secondary antibody–dye conjugates, anti-rabbit AF647 (Thermo Fisher), for 40 min, washed thoroughly with PBS three times at room temperature and stored at 4°C. Upon this step, the staining for the first target (H3K9me3) is finished. The sample can be imaged for single color on heterochromatic target H3K9me3 or incubated overnight in blocking buffer with serum (90% of the blocking buffer mentioned above + 10% goat serum) for the following 2-color and 3-color staining.
4. After overnight blocking step, the sample would go through a similar process as Step 3. But the primary incubation and secondary incubation time are both 1 hour. The blocking buffer will be modified to blocking buffer with serum (90% of the blocking buffer mentioned above + 10% goat serum) and the washing buffer to washing buffer with serum (99% of the blocking buffer mentioned above + 1% goat serum) The primary antibody is rat anti-RNA Polymerase II (Abcam) and the secondary antibody is goat anti-rat AF488 (Abcam). Upon the finish of the RNA Polymerase II staining, the sample can be imaged for 2-color on heterochromatin target H3K9me3 and functional target Polymerase II or incubated overnight in blocking buffer with serum for 3-color staining
5. After overnight blocking step, the sample would go through a similar process as Step 3. But the primary incubation and secondary incubation time are both 1 hour. The blocking buffer will be modified to blocking buffer with serum (90% of the blocking buffer mentioned above + 10% goat serum) and the washing buffer to

washing buffer with serum (99% of the blocking buffer mentioned above + 1% goat serum) The primary antibody is mouse anti-H3K27ac (Thermofisher) and the secondary antibody is goat anti-rat AF568 (Abcam). Upon the finish of the RNA Polymerase II staining, the sample can be imaged for 2-color on heterochromatin H3K9me3, functional target Polymerase II and euchromatin target H3K27ac.
